# Cycle oxidation behavior and anti-oxidation mechanism of hot-dipped aluminum coating on TiBw/Ti6Al4V composites with network microstructure

**DOI:** 10.1038/s41598-018-24242-0

**Published:** 2018-04-10

**Authors:** X. T. Li, L. J. Huang, S. L. Wei, Q. An, X. P. Cui, L. Geng

**Affiliations:** 1 State Key Laboratory of Advanced Welding and Joining, Harbin Institute of Technology, P.O. Box 433, Harbin, 150001 P.R. China; 2 Key Laboratory of Advanced Structural-Functional Integration Materials & Green Manufacturing Technology, School of Materials Science and Engineering, Harbin Institute of Technology, Harbin, 150001 P.R. China; 3 Present Address: Department of Materials Science and Engineering, Massachusetts Institute of Technology, 77 Massachusetts Avenue, Cambridge, MA 02139 USA

## Abstract

Controlled and compacted TiAl_3_ coating was successfully fabricated on the network structured TiBw/Ti6Al4V composites by hot-dipping aluminum and subsequent interdiffusion treatment. The network structure of the composites was inherited to the TiAl_3_ coating, which effectively reduces the thermal stress and avoids the cracks appeared in the coating. Moreover, TiB reinforcements could pin the TiAl_3_ coating which can effectively improve the bonding strength between the coating and composite substrate. The cycle oxidation behavior of the network structured coating on 873 K, 973 K and 1073 K for 100 h were investigated. The results showed the coating can remarkably improve the high temperature oxidation resistance of the TiBw/Ti6Al4V composites. The network structure was also inherited to the Al_2_O_3_ oxide scale, which effectively decreases the tendency of cracking even spalling about the oxide scale. Certainly, no crack was observed in the coating after long-term oxidation due to the division effect of network structured coating and pinning effect of TiB reinforcements. Interfacial reaction between the coating and the composite substrate occurred and a bilayer structure of TiAl/TiAl_2_ formed next to the substrate after oxidation at 973 K and 1073 K. The anti-oxidation mechanism of the network structured coating was also discussed.

## Introduction

Titanium matrix composites (TMCs) possessed superior characteristics such as the low density, good mechanical properties and high-temperature durability^1^. Specially, the TMCs with network distribution of reinforcements fabricated by Huang *et al*.^2^ exhibited superior mechanical properties compared to the conventional composites with homogeneous microstructure^3–7^, which is potential candidate for further application in the fields of aerospace, military and commercial automobile. However, the applications of the TMCs at high temperatures have been seriously restricted due to their poor oxidation resistance^8–12^. Hence, it is a subject concern to improve the oxidation resistance of the TMCs without changing the bulk properties. Surface coating is an alternative method to improve the oxidation resistance of the TMCs. Several coatings such as aluminide^13–15^, Ti–Al–X (X = Cr, Nb)^16,17^, MCrAlY-type^18,19^ and silicide/ceramics^20–22^ were fabricated on the Ti-based alloy surface, which can provide protection against the oxidation. Among these coatings, aluminide coating which could form a protective Al_2_O_3_ scale had been widely applied because of the practical superiority over others.

The TiAl_3_ coating and the Pt-modified coating prepared by packing aluminizing process improved the oxidation resistance of Ti-based IMI-834 alloy under high temperature^23,24^ and the effect of through-thickness cracks on long-time oxidation properties of the coating was also discussed^25^. The studies by Zhou *et al*.^26,27^ showed that the Al-Si coating produced by low oxygen pressure fusing technology provides good protection against oxidation and the prolong fuse time was adverse to the oxidation resistance. The interrupted and isothermal oxidation behaviors of the hot-dipped aluminum coatings on Ti6Al4V alloy^28^ showed the TiAl_3_ coating markedly decreased the oxidation rate compared to the alloy. Jeng^29^ further investigated the oxidation behavior and microstructure evolution of the hot-dipped aluminum coating at 1073 K for different isothermal oxidation time. However, the aluminide coatings formed on Ti alloy surface are liable to make cracks after long-time oxidation due to the great thermal stress of the coatings.

According to the previous investigation^30^, the network structured TiBw/Ti6Al4V composites showed superior high-temperature mechanical properties, but its oxidation resistance is destructed due to the strong affinity of Ti alloy towards oxygen and increased interface between reinforcement and matrix^31^. However, there is no study reported about the oxidation resistance of coating on the TMCs up to now. Hence, it is very meaningful to investigate coating of the TiBw/Ti6Al4V composites. Moreover, the network structure distribution of the TiB reinforcements maybe beneficial to improve the oxidation resistance of the coatings. The objective of the present work was to investigate the high temperature oxidation behavior and anti-oxidation mechanism of the hot-dipped aluminum coating fabricated on the network structured TiBw/Ti6Al4V composites.

## Results and Discussion

### Microstructure of the hot-dipped coating

**Figure 1:**
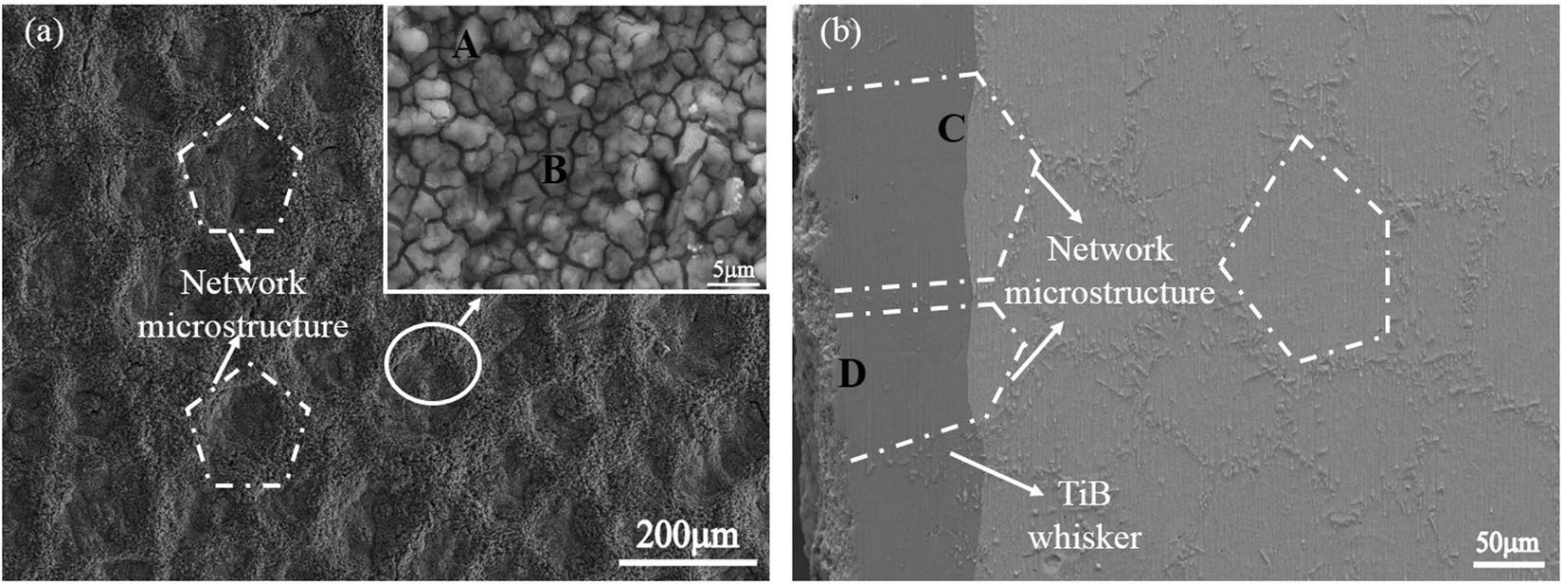
The SEM images of the coating on TiBw/Ti6Al4V composites (**a**) the surface image (**b**) the cross-section image.

After hot-dip aluminizing and interdiffusion treatment, a surface coating was produced on the TiBw/Ti6Al4V composite substrate. The surface morphology and the cross-section image of the coating are shown in Fig. 1. The Fig. 1(a) with the EDS analysis shows a network structured TiAl_3_ coating was successfully fabricated on the TiBw/Ti6Al4V composite substrate, this is due to the network structure distribution of the TiB reinforcements in the composites. The thermal expansion coefficients of Ti6Al4V alloy were 9.0 × 10^−6^K^−1^ at 573 K and 10.0 × 10^−6^ K^−1^ at 773 K measured by Zhang *et al*.^32^. There are no available experimental values for TiAl_3_, but the theoretical value of 15.0 × 10^−6^ K^−1^ at 293 K was reported in previous study^33^. Hence, there were cracks generated in the coating by the mismatch of thermal expansion coefficient between the TiAl_3_ coating and Ti6Al4V alloy reported in the previous article^28^. According to the rule of mixture, the thermal expansion coefficient of TiBw/Ti6Al4V is very similar to the value of Ti6Al4V. In our study, the network structure of the coating actually divided the whole coating into many network cells with small size, and this division can effectively reduce the thermal stress caused by the mismatch in thermal expansion coefficient between the TiAl_3_ coating and the composite substrate, which avoid the existence of cracks in the TiAl_3_ coating.

**Figure 2:**
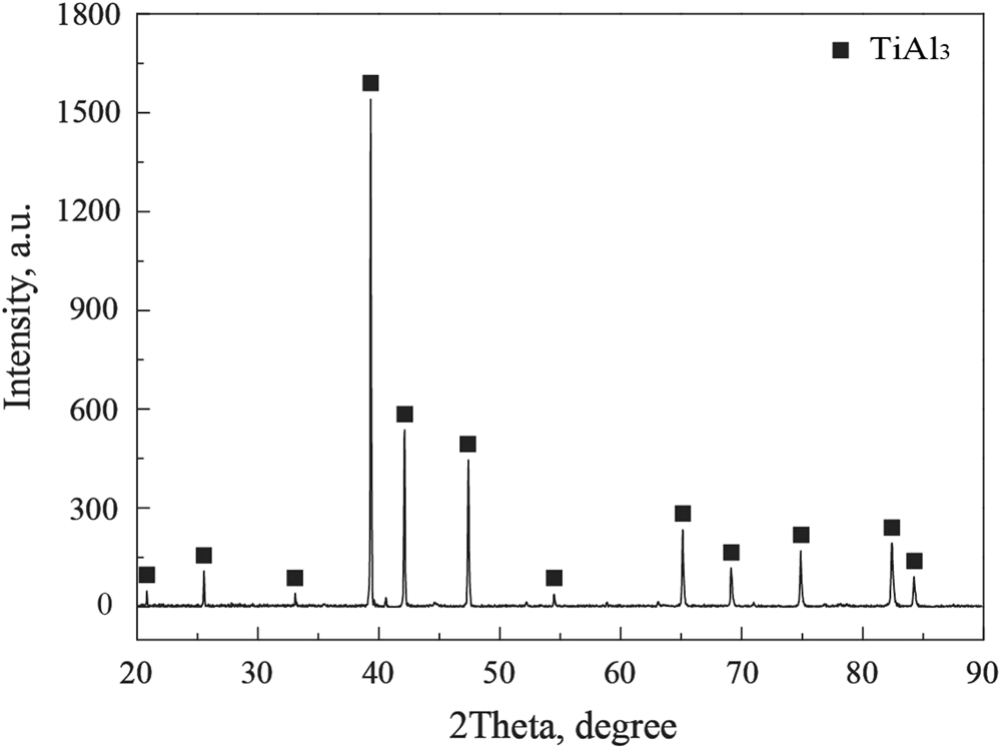
The surface XRD pattern of the coating on TiBw/Ti6Al4V composites.

From the Fig. 1(b), it can be seen that the average thickness of the coating is about 95 μm. It is worth notice that there are TiB whiskers inserting into the TiAl_3_ coating, and the TiB whiskers in the coating still maintains the network structure, which can effectively improve the bonding strength of the coating and intensify the effect of network structure. In addition, it can also be found the interface between the coating and the TiBw/Ti6Al4V composite substrate is not smooth, and the interfaces of TiB whiskers located are more inward diffusion than other locations, this is mainly due to the different diffusion methods of Al atom and Ti atom in the Ti6Al4V substrate and TiB whisker location. The diffusion method in Ti6Al4V substrate is mainly bulk diffusion, and in the location of TiB whisker is bulk diffusion and interface diffusion, which leads to the faster diffusion speed in the location of TiB whisker. This is the reason for the network structure formation of the TiAl_3_ coating. A compacted coating was formed and there are no pores and cracks in the coating, which is attributed to the division effect of the network structure and the pinning effect of the TiB whiskers. The surface XRD pattern of the coating after the hot-dip aluminizing and treatment is shown in Fig. 2. Only TiAl_3_ diffraction peaks are detected, which indicates that only TiAl_3_ is formed on the TiBw/Ti6Al4V composites. This phenomenon is further confirmed by the SEM results as shown in Fig. 2 and Table 1.

**Table 1:** Chemical compositions of the coating marked in Fig. 2 (at%, EDS).

Point	Ti	Al
A	25.60	74.40
B	26.01	73.99
C	24.39	75.61
D	25.49	74.51

### Oxidation kinetics of the coated and uncoated TiBw/Ti6Al4V composites

**Figure 3:**
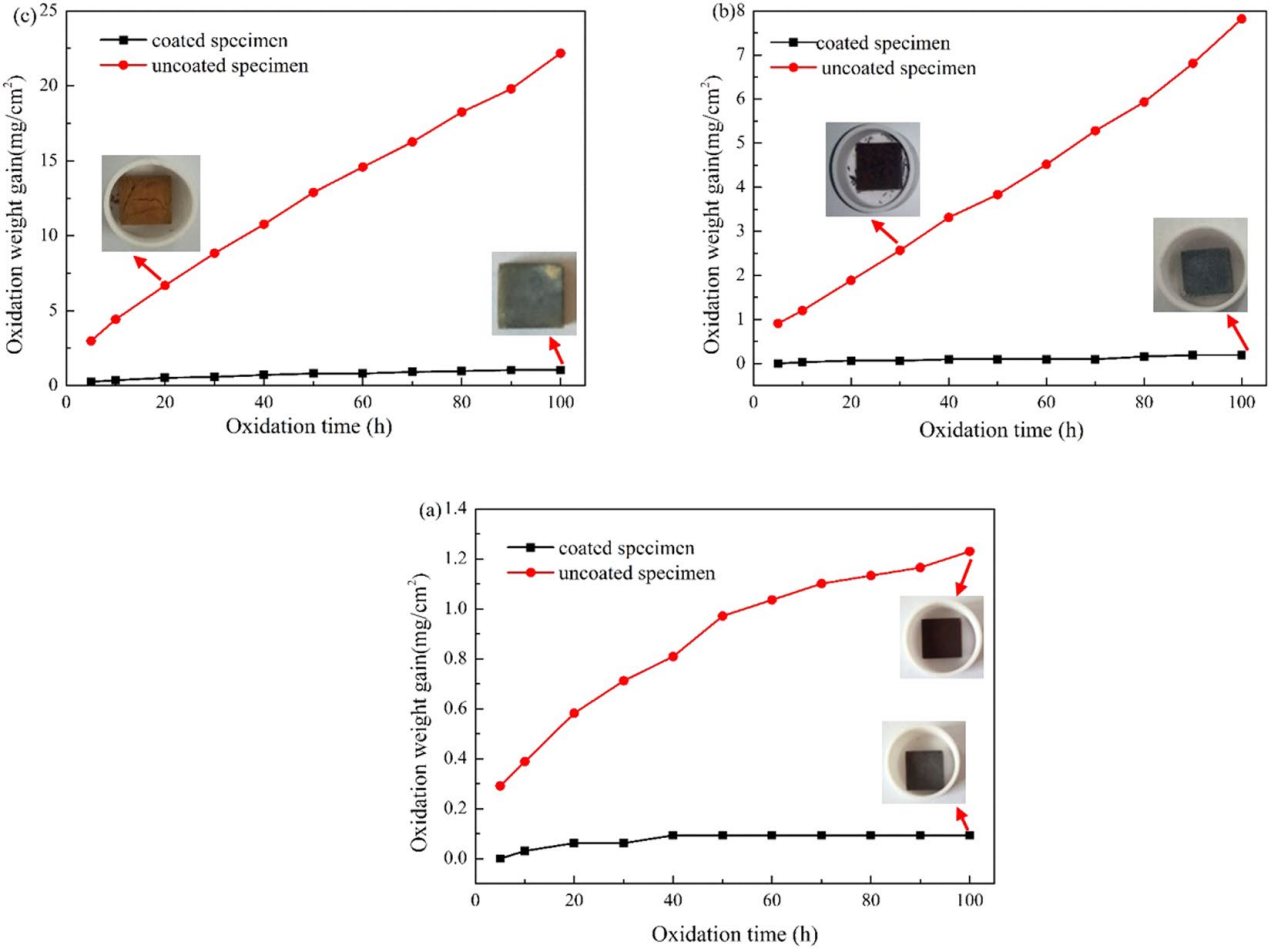
The cycle oxidation kinetic curves of the coated and uncoated TiBw/Ti6Al4V composite specimens (**a**) 873 K, (**b**) 973 K, (**c**) 1073 K.

The cycle oxidation kinetic curves of the coated and uncoated specimens at 873 K, 973 K and 1073 K in air are shown in Fig. 3. It is obvious that the oxidation rate of the TiAl_3_ coated composites significantly reduces compared to the TiBw/Ti6Al4V composites under high temperatures, especially for 973 K and 1073 K. The oxide scales of both the TiAl_3_ coated and uncoated TiBw/Ti6Al4V composites don’t spall and their oxidation kinetic curves obey parabolic law at the temperature of 873 K. Moreover, the mass gain of the coated and uncoated composites are 0.09 mg/cm^2^ and 1.23 mg/cm^2^ after 100 h, respectively, implying the TiAl_3_ coating has little oxidation and the degree of oxidation for the TiBw/Ti6Al4V composites is not very severe at 873 K for 100 h. When the temperature reaches 973 K and 1073 K, the cycle oxidation kinetic curves of the coated composites still follow the parabolic law but the uncoated composites follow the linear law. It was well known the reason of the parabolic law is that oxide scales prevent oxidation and reduce the oxidation rate of the substrate, and the linear law is due to the non-protective oxide product is formed which can’t protect the substrate. The oxide scales of the uncoated composites start to partially spall at 30 h for 973 K and at 20 h for 1073 K, respectively, however the oxide scales of the coated composites still haven’t any spallation phenomenon. The mass gain of the coated composites is 0.19 mg/cm^2^ and 1.04 mg/cm^2^ for 100 h at 973 K and 1073 K, and that of the uncoated composites is 7.21 mg/cm^2^ and 22.17 mg/cm^2^, respectively. It can also be found that the oxidation rate of the TiBw/Ti6Al4V composites quickly increases with increasing temperature, but the oxidation rate of the coated composites is very slowly increased with increasing temperature. Therefore, it can be concluded that the TiAl_3_ coating can significantly improve the oxidation resistance of the TiBw/Ti6Al4V composites under high temperatures.

### Surface morphology of the oxidized TiBw/Ti6Al4V composites

**Figure 4:**
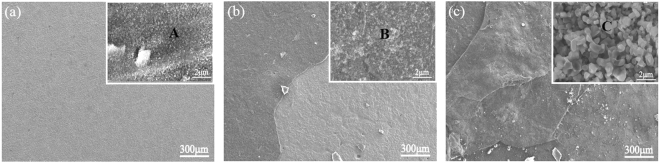
The surface morphology of the TiBw/Ti6Al4V composites oxidized at different temperatures for 100 h.

**Figure 5:**
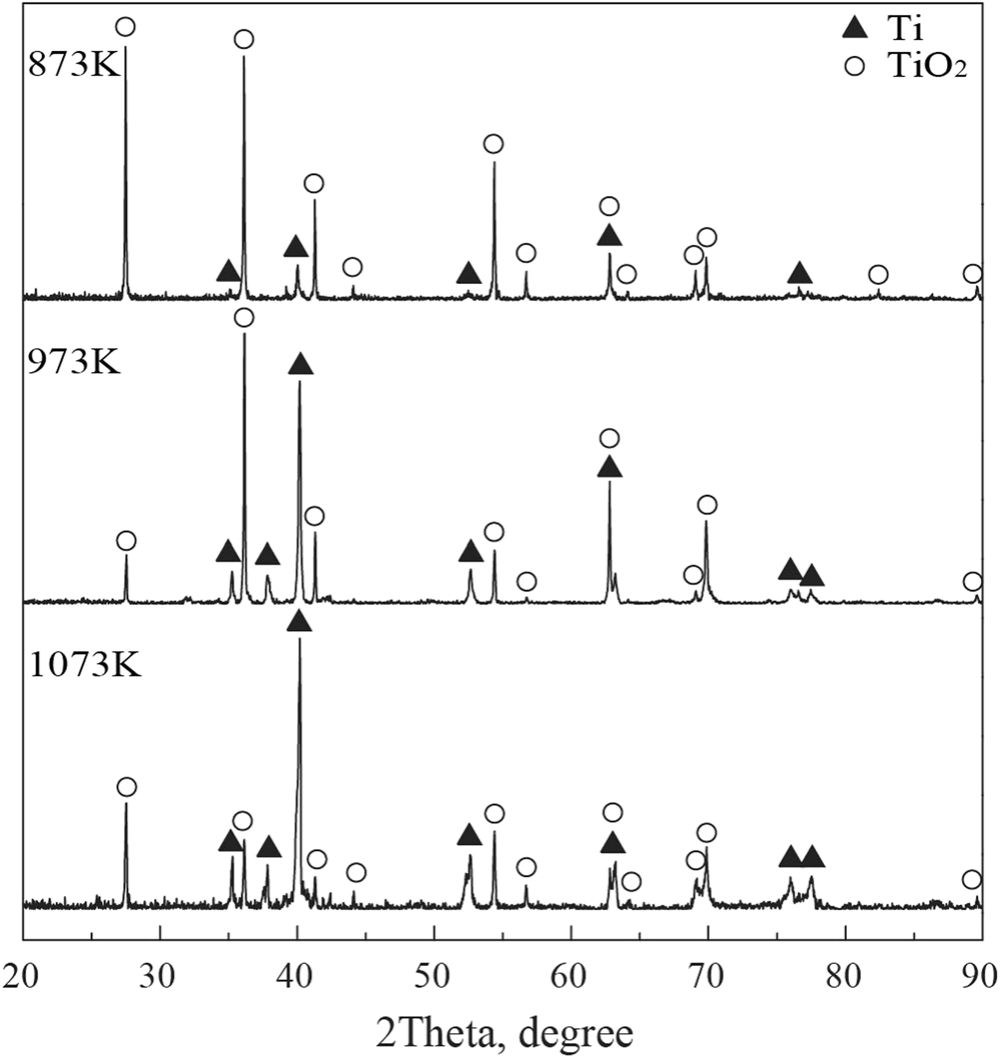
The XRD patterns of oxide scales of the TiBw/Ti6Al4V composites in different temperatures for 100 h.

Figure 4 shows the surface morphology of the TiBw/TC4 composites oxidized at different temperatures for 100 h. As shown in Fig. 5(a), it’s obvious that the oxide scale don’t spall during the oxidation process, indicating the degree of oxidation of the composites is not severe at 873 K for 100 h. When the temperature reaches 973 K, the oxide scales spallation happened, implying the oxidation degree is very serious at 973 K. Moreover, it can be also seen the spallation degree of oxide scales is more serious at 1073 K, which means that the oxidation resistance of the composites is the worst at 1073 K. Combining with the composition analysis of points listed in Table 2, it can be concluded the TiO_2_ oxide particles were formed on the composites. From the enlarged images in Fig. 4, it can be seen that the size of TiO_2_ oxide particles increases with increasing temperature, which could further explain the oxidation kinetic law of the composites under high temperature. The very tiny TiO_2_ oxide particles are formed at the temperature of 873 K, and the oxide film is very thin that it wouldn’t spall during the oxidation process, so the oxidation degree of the composites is not serious, which is consistent with the results of the parabolic law of the oxidation kinetic curve in Fig. 3(a). The sizes of TiO_2_ oxide particles begin to grow up when the temperature reaches 973 K, and the TiO_2_ oxide scales formed on the surface of composites become thick and it can’t protect the composite substrate, the protective Al_2_O_3_ oxide film also can’t be formed owing to the low content of Al in the composites. Hence, the spallation of thick non-protective oxide scales would happen at 973 K, and the fresh exposed surface would continue to the oxidation process again, so the oxide scales have a multilayer spallation phenomenon at 973 K for 100 h, that is why the cycle oxidation kinetic curve of the composites follows the linear law at 973 K as shown in Fig. 3(b). The sizes of TiO_2_ oxide particles have a drastic growth during oxidation process at 1073 K, the oxide scales become more thick and it would spall more easily, so the multilayer spallation of oxide scales is more serious, which is consistent with the results obtained by the oxidation kinetic curves in Fig. 3. The XRD patterns of the oxide scales on TiBw/Ti6Al4V composites in different temperatures for 100 h are displayed in Fig. 5. It can be seen that only TiO_2_ oxide detected on the surface of the composites under high temperatures oxidation for 100 h, which further explains the poor oxidation resistance of the composites during the high temperature oxidation process.

**Table 2:** Composition analysis of different points of oxidized TiBw/Ti6Al4V composites marked in Fig. 5 (at%, EDS).

Point	Ti	Al	O
A	34.41	1.26	64.33
B	33.21	1.01	65.78
C	25.26	1.52	73.22

### Surface morphology and microstructure of the oxidized coating

**Figure 6:**
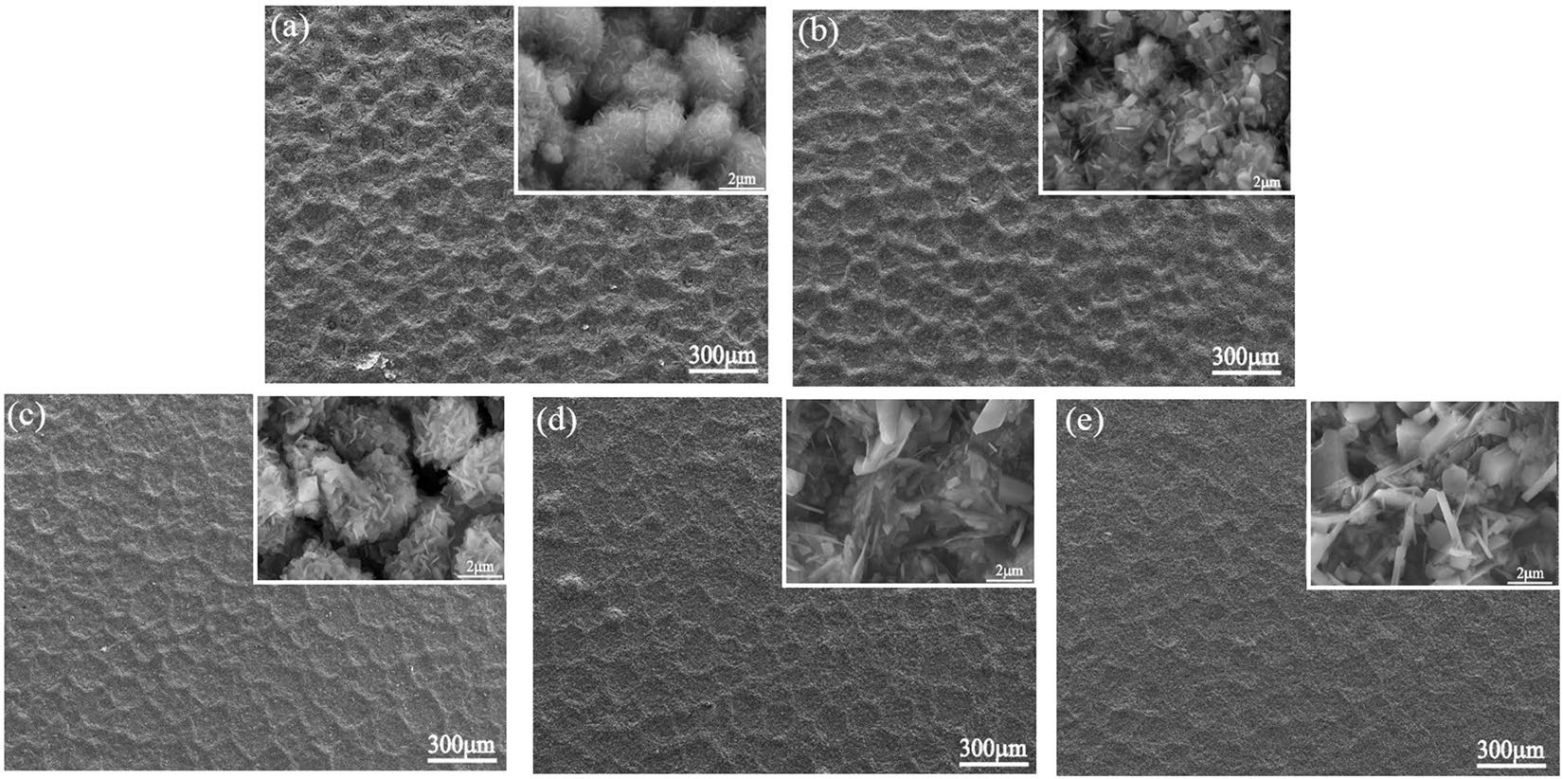
The morphology of the oxidized surface on TiAl_3_ coating at 873 K for 100 h (**a**), 973 K for 100 h (**b**), 1073 K for 5 h (**c**), 1073 K for 50 h (**d**), 1073 K for 100 h (**e**).

Figure 6 displays the SEM morphologies of the oxidized surface of the TiAl_3_ coated TiBw/Ti6Al4V composites at different temperatures for different oxidized times. As seen in Fig. 6(a,b and e), it can be observed that the network structured oxide scales were formed after 100 h oxidation. It is clearly seen the thickness of oxide film with network structure gradually increases with increasing temperature, and there is no oxide scale spallation happened. Further observation in Fig. 6(a) shows the TiAl_3_ particles produce slightly oxidation, and the tiny Al_2_O_3_ oxides in the shape of whisker are generated on the TiAl_3_ particles. Meanwhile, it can be also seen that the Al_2_O_3_ oxides have different orientation which means the growth of the Al_2_O_3_ oxides possess preferential growth orientation. In addition, it is worth noting that both the sizes and the quantities of Al_2_O_3_ have obvious change with increasing temperature. When the coating is oxidized at 973 K, the quantities of the Al_2_O_3_ increase obviously and the size has grown to some extent, indicating the oxidation degree of the coating is more serious compared to that at 873 K.The size of Al_2_O_3_ has drastic growth condition when the oxidation temperature rises up to 1073 K, which is in good agreement with the results obtained by the cycle oxidation kinetic curves in Fig. 3.

It can be seen from Fig. 6(c–e) that the oxide scales with network structure on the surface of the TiAl_3_ coating become more and more thick as the oxidation time prolong at 1073 K. At the beginning of the oxidation process, there are also Al_2_O_3_ oxides with the shape of whisker produced on the TiAl_3_ particles, and the preferential growth orientation of Al_2_O_3_ oxides still maintain. Further observation displays the sizes of Al_2_O_3_ oxides are bigger than these oxidized at 873 K for 100 h, which suggests the temperature on oxidation resistance of the coating is more serious than the oxidation time. The drastic growth phenomenon of partial oxide has taken place in the middle period of oxidation at 1073 K, implying the oxidation condition is more serious compared to that at 973 K for 100 h. When the oxidation time prolong to 100 h, the Al_2_O_3_ oxides size has further grown, but the oxide film still keeps the network structure and don’t have any spallation happened, implying the TiAl_3_ coating still possesses better oxidation resistance at 1073 K, and this is in good agreement with the results by the cycle oxidation kinetic curves in Fig. 3(c). This can be attributed to that the network structure can reduce the thermal stress and the pinning effect of TiB whiskers can improve the bonding strength.

**Figure 7:**
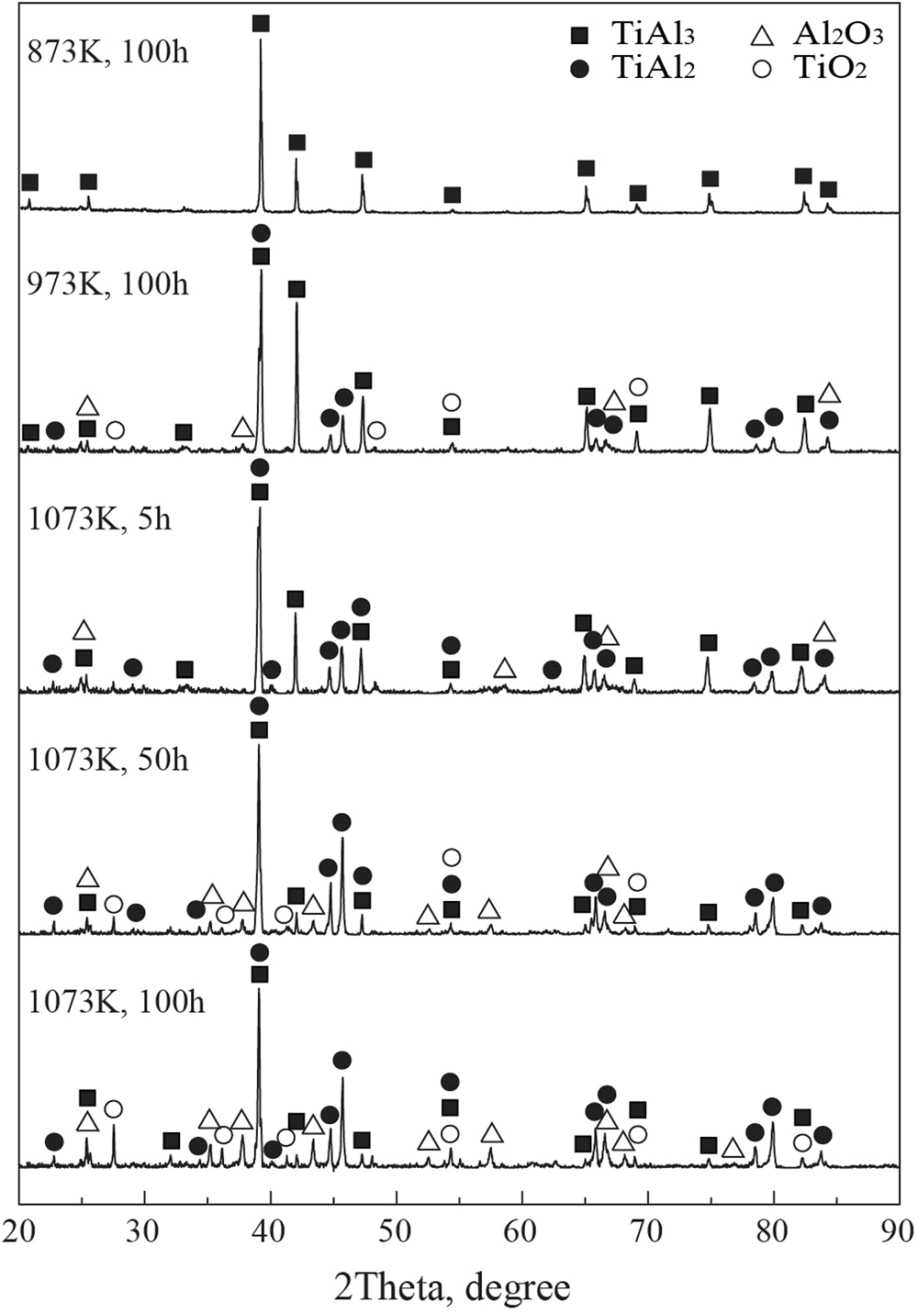
The XRD patterns of oxide scales of TiAl_3_ coating at different temperatures for different times.

The XRD patterns of the oxide scales on TiAl_3_ coating oxidized in different temperatures for different times are displayed in Fig. 7. It can be seen from Fig. 7(a) that only TiAl_3_ existed on the surface of coating oxidized in 873 K for 100 h, this is ascribed to the very slight oxidation occurred on the coating, and the quantity of the oxides is so few that it can’t be detected by XRD, which further confirm the conclusion that the TiAl_3_ coating have very good oxidation resistance at 873 K. In addition, it can be noted that there are a large number of TiAl_3_ and a few of TiAl_2_ and Al_2_O_3_ on the surface in the early stage of oxidation at 1073 K, this is mainly due to the reaction 2TiAl_3_ + 3/2O_2_ = 2TiAl_2_ + Al_2_O_3_ happened between the TiAl_3_ and O_2_ in the oxidation process^34^, suggesting the oxidation of coating become serious compared to that at 873 K, which is consistent with the analysis results by Figs 3 and 6.

Further observation shows that a few of TiO_2_ can be detected with the increase of oxidized time, which is due to an amount of O_2_ inward diffuse continuously during the oxidation process in long term, the TiAl_2_ and O_2_ make further reaction TiAl_2_ + 5/2O_2_ = Al_2_O_3_ + TiO_2_. Therefore, it’s reasonable to assume that a mixture of Al_2_O_3_ + TiO_2_ was formed below the dense and continuous Al_2_O_3_ oxide film, because the dense Al_2_O_3_ layer has good protection for the coating in long time oxidation, hence the TiAl_3_ coating still has good oxidation resistance at 1073 K. Similar oxidation products are detected on the surface at 973 K for 100 h, but the diffraction peaks intensities and quantities of Al_2_O_3_ and TiO_2_ reduce obviously compared to those at 1073 K for 100 h, which further indicates that the degree of oxidation on TiAl_3_ coating gradually becomes serious with increasing temperature, this is in good agreement with the results obtained by the cycle oxidation kinetic curves and surface morphologies analysis.

**Figure 8:**
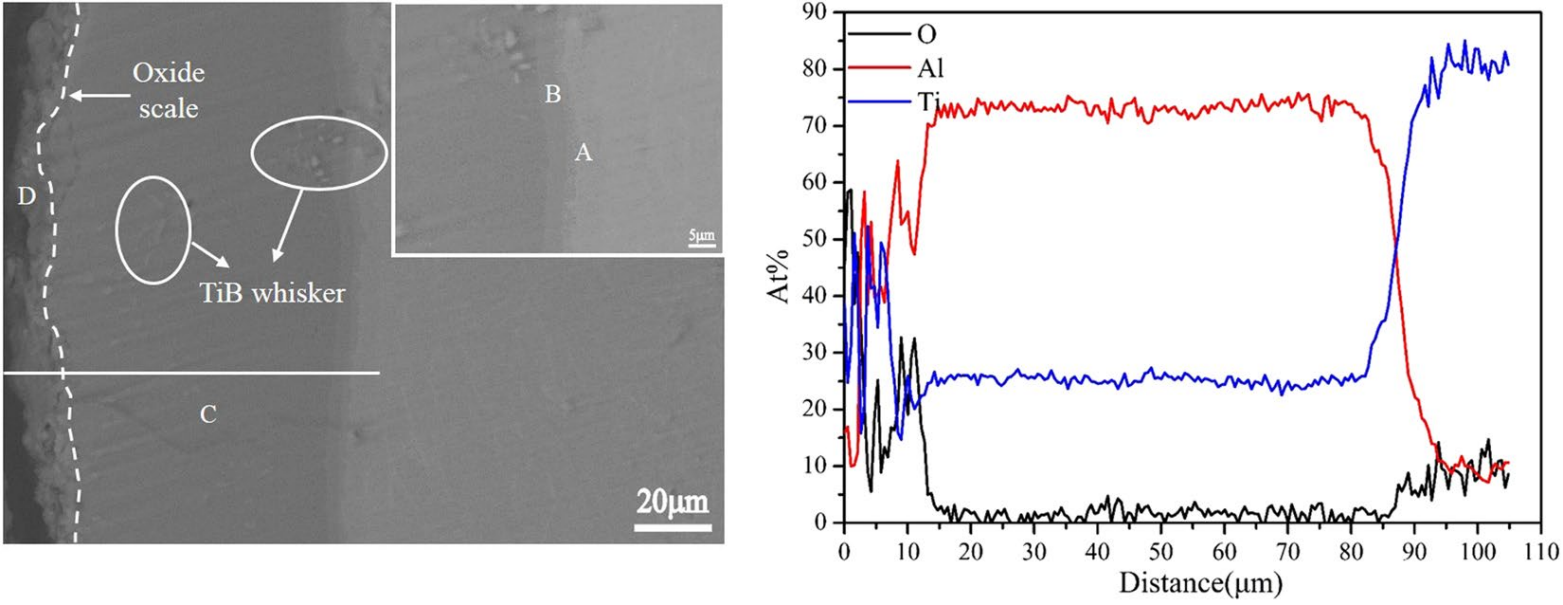
The cross-section morphology and EDS line scan of TiAl_3_ coating after 100 h cycle oxidation at 973 K.

The cross-sectional microstructure of the TiAl_3_ coating after 100 h oxidation at 973 K is displayed in Fig. 8. The oxidized coating becomes a multilayer structure and still has good adhesion to the TiBw/Ti6Al4V composite substrate. It is noticeable that the oxidized coating is still free of cracks and different with the previous results^23,25,27–29^, which is attributed to the network structure of the coating after oxidation. The BSE image shows that the oxidized coating divides into four different layer, according to the line scan in Fig. 8 and EDS analysis in Table 3, it can be concluded that the oxide film about 12 μm was formed in the outmost layer of the oxidized coating, and the oxide film should consist of a great of Al_2_O_3_ and a few TiO_2_. According to the EDS analysis of point C listed in Table 3, the composition of the layer next to oxide film is still TiAl_3_, indicating the oxide scales effectively keep O element from entering into the coating, which confirms the good oxidation resistance of the coating at 973 K for 100 h. Furthermore, the interfacial reaction zone with layered structure about 5 μm is observed between the TiAl_3_ coating and composite substrate as shown in the insert image. The layer next to composite substrate is made up of TiAl and the other layer is composed of TiAl_2_ according to the EDS analysis results of points A and B, this is mainly due to diffusion reaction of Ti atom and Al atom in the interface between the TiAl_3_ coating and TiBw/Ti6Al4V composite substrate.

**Table 3:** Composition analysis of different points of the oxidized TiAl_3_ coating marked in Fig. 9 (at%, EDS).

Point	Ti	Al	O
A	47.50	52.50	0
B	35.27	64.73	0
C	26.09	73.91	0
D	8.11	36.07	54.82

**Figure 9:**
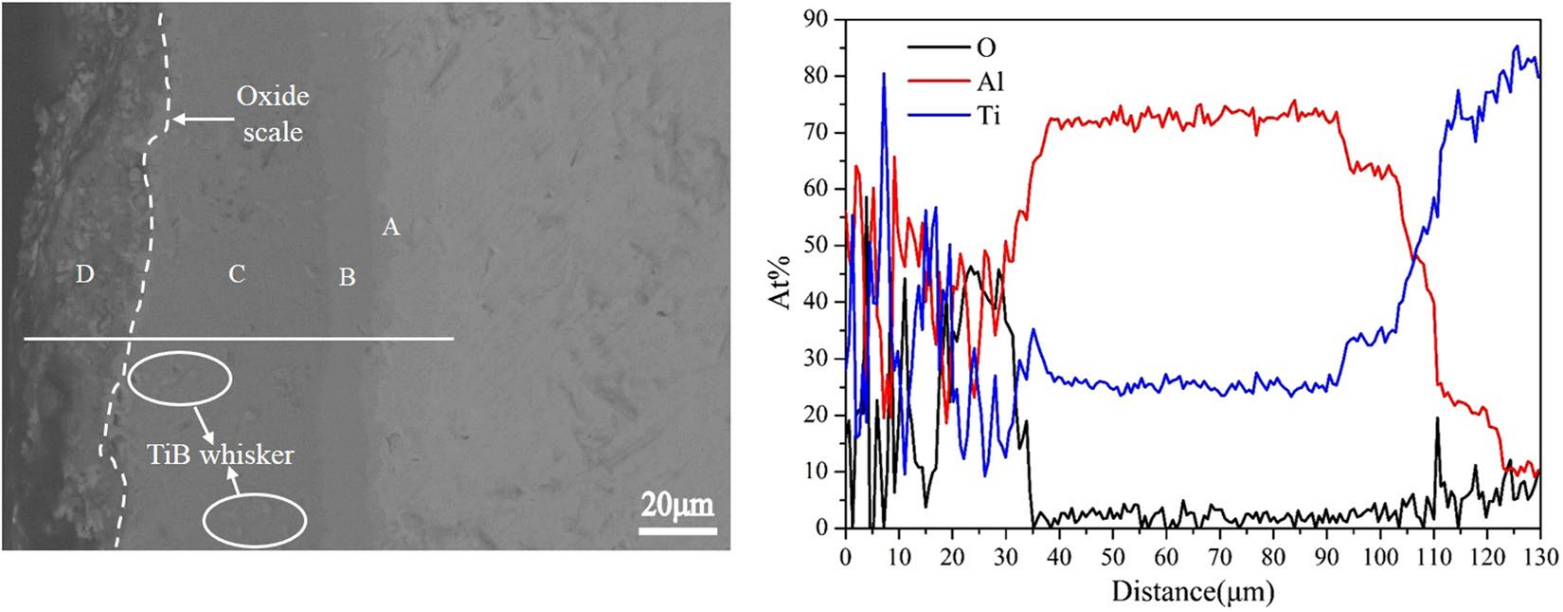
The morphology of cross-section and EDS line scan on the TiAl_3_ coating after 100 h oxidation at 1073 K.

**Figure 10:**
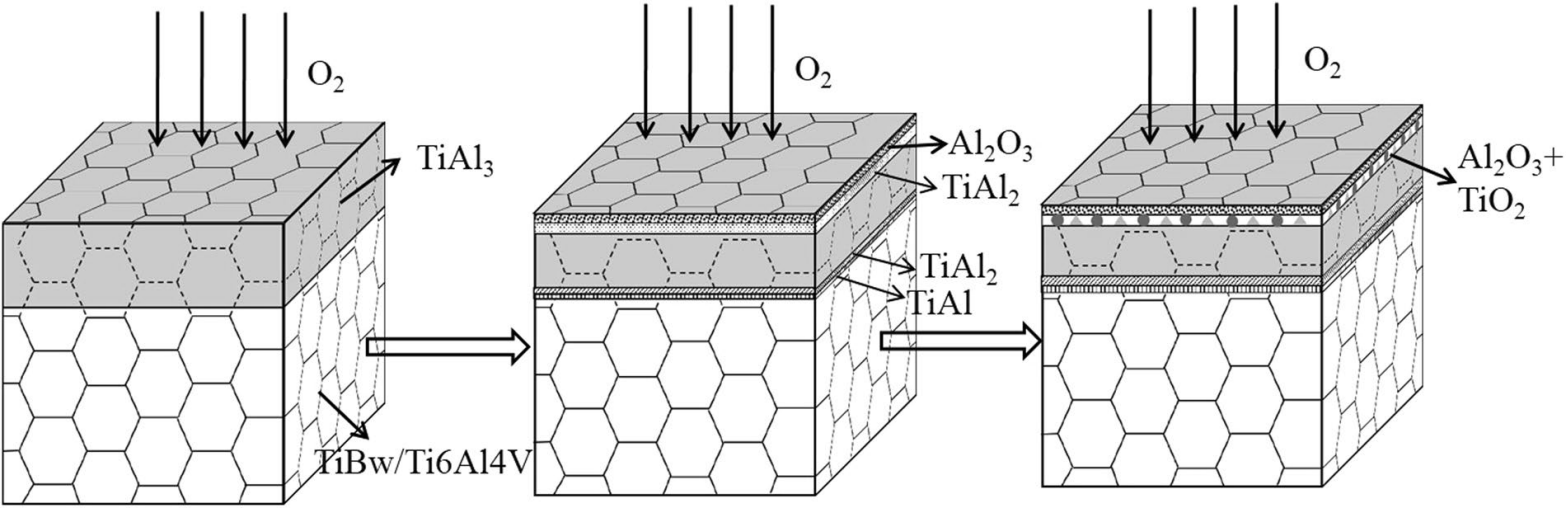
The schematic diagram of oxidation process of the TiAl_3_ coated TiBw/Ti6Al4V composites under high temperature.

Figure 9 displays the cross-section image of the TiAl_3_ coating oxidized at 1073 K for 100 h. It is evident that the oxidized coating morphology is similar to that at 973 K and still divides into four different layers. The whole oxidized coating also keeps good adhesive to the composite substrate. Moreover, there are also no cracks appeared, which is mainly due to that the oxidized coating at 1073 K for 100 h still maintains the network structure as shown in Fig. 6(e). It can be clearly seen that the thickness of the outmost oxide scale is about 33 μm, and the oxide scale also effectively prevents O element into the coating according to the line scan in Fig. 10 and EDS analyze results of points C and D in Table 4. It can be concluded that the coating still maintains the good protection for the composite substrate at 1073 K, but the oxidation is more serious than that at 973 K. In addition, it is also observed that the thickness of interface reaction zone which is made up of TiAl_2_ and TiAl layer is increased to 15 μm. Therefore, it is reasonable to deduce that TiAl_3_ coating is mainly consumed by the oxidation reaction in the outmost layer and interface reaction in the interface between the coating and the composite substrate during the high temperature oxidation. Owing to the faster outward diffusion speed of Al atom compared to Ti atom and the high content of Al atom in the coating, the continuous and dense oxide scales can be formed during the high temperature oxidation, which can effectively protect the composites against the oxidation. The outward diffusion of Ti atom in the composite substrate can lead to the diffusion reaction in the interface between the coating and composite substrate, so the TiAl_2_ and TiAl layer can be formed in the interface during the high temperature oxidation. Moreover, it can be observed that the thickness of oxidation layer and interface reaction zone at 1073 K is bigger than that at 973 K, which is mainly ascribe to the diffusion rates of Ti atom and Al atom increase with increasing temperature.

**Table 4:** Composition analysis of different points of the oxidized TiAl_3_ coating marked in Fig. 10 (at%, EDS).

Point	Ti	Al	O
A	52.22	47.78	0
B	33.81	66.19	0
C	25.93	74.07	0
D	9.67	24.97	65.36

### Mechanism of anti-oxidation

Figure 10 shows the high temperature oxidation schematic diagram of the TiAl_3_ coated TiBw/Ti6Al4V composites. The TiB reinforcements with network distribution are inserted the coating, which can effectively improve the bonding strength between the coating and composite substrate and divide the whole coating into small network cells, moreover, the network structure is also inherited to the dense and continuous oxide scale of Al_2_O_3_. The spallation of oxide scale is an important factor that can affect the cycle oxidation kinetics and microstructure evolution, and it often induces through the thermal stress caused by the mismatch of thermal expansion coefficient between the oxide scale and the coating. Because the thermal stress is difficult to measure by experiment method, and it can be predicted through a dual thin-scale model proposed by Timoshenko^35^, and the formula can be expressed as:

1 $${\sigma }_{O{\rm{x}}}=\frac{-({\alpha }_{Ox}-{\alpha }_{M}){\rm{\Delta }}T}{\frac{2{\delta }_{Ox}(1-{\mu }_{M})}{{\delta }_{M}{E}_{M}}+\frac{(1-{\mu }_{Ox})}{{E}_{Ox}}}$$

where *α*_*Ox*_, *α*_*M*_, *δ*_*Ox*,_ *δ*_*M*_, *μ*_*Ox*_, *μ*_*M*_, *E*_*Ox*_, *E*_*M*_ and Δ*T* represent the thermal expansion coefficient, thickness, Poisson ratio, Young’s modulus of the oxide scale and coating and the temperature difference between oxidation and room temperature, respectively.

Hence, it is reasonable to assume the thermal stress between the oxide scale and the coating is in direct proportion to the difference values of thermal expansion coefficients between the coating and oxide scale. Owing to the insertion of TiB whisker, the network structured oxide scale and TiAl_3_ coating can be supposed as the composite of the oxide scale and TiB, the TiAl_3_ and TiB, respectively. The thermal expansion coefficients of the composites can be empirically estimated by the Turner’s model^36^, which is expressed as follows:

2 $${\alpha }_{c}={\alpha }_{m}\cdot {V}_{{\rm{m}}}+{\alpha }_{p}\cdot {V}_{p}+{V}_{p}\cdot {V}_{m}\cdot ({\alpha }_{p}-{\alpha }_{m})\cdot ({K}_{p}-{K}_{m})/[{V}_{m}\cdot {K}_{m}+{V}_{p}\cdot {K}_{p}+3(\frac{{K}_{p}\cdot {K}_{m}}{4{G}_{m}})]$$

where *K*_*i*_, *V*_*i*_, *G*_*i*_ and *α*_*i*_ are the bulk modulus, volume fraction, shear modulus and thermal expansion coefficient for different phase. The parameters used in the calculations are summarized in Table 5. The calculated thermal expansion coefficients of network structured Al_2_O_3_ oxide scale and TiAl_3_ coating is 6.69 K^−1^ and 14.68 K^−1^, respectively, and the difference value between the network structured oxide scale and coating becomes smaller compared to that in the whole structure. It can be concluded that the network structure by the insertion of TiB whiskers can reduce the thermal stress between the oxide scale and coating, which further decrease the tendency of cracking even spalling about oxide scale. Therefore, the Al_2_O_3_ oxide scales with network structure can effectively prevent the O element into the coating and have a good protection for the composites during the cycle oxidation process.

**Table 5:** Parameters used to calculated the thermal expansion coefficients of composites^33,37–39^.

Materials	*α*, 10^−6^ K^−1^	*K*, GPa	*G*, GPa
Al_2_O_3_	6.6	247	155
TiAl_3_	15	105	93
TiB	8.6	206	184

The thermal expansion coefficients of titanium aluminum compound decrease with the decrease of Al composition in the Ti-Al system^33^. Therefore, the formation of TiAl_2_ beneath the oxide scales also can reduce properly the thermal stress between the oxide scales and coating, which is contributed to improving the bonding strength of oxide scales to the coating. In addition, there is reaction zone composed of TiAl layer and TiAl_2_ layer existed in the interface between the coating and composite substrate, this is mainly due to the interdiffusion reaction of Ti atom and Al atom in the interface during the high temperature oxidation process. The bilayer structure can decrease thermal stress between the coating and composite substrate, which is beneficial to avoid the cracks appeared in the coating during the oxidation process. In conclusion, the network structured TiAl_3_ coating provides very good protection for the TiBw/Ti6Al4V composites during the high temperature oxidation process.

## Methods

**Figure 11:**
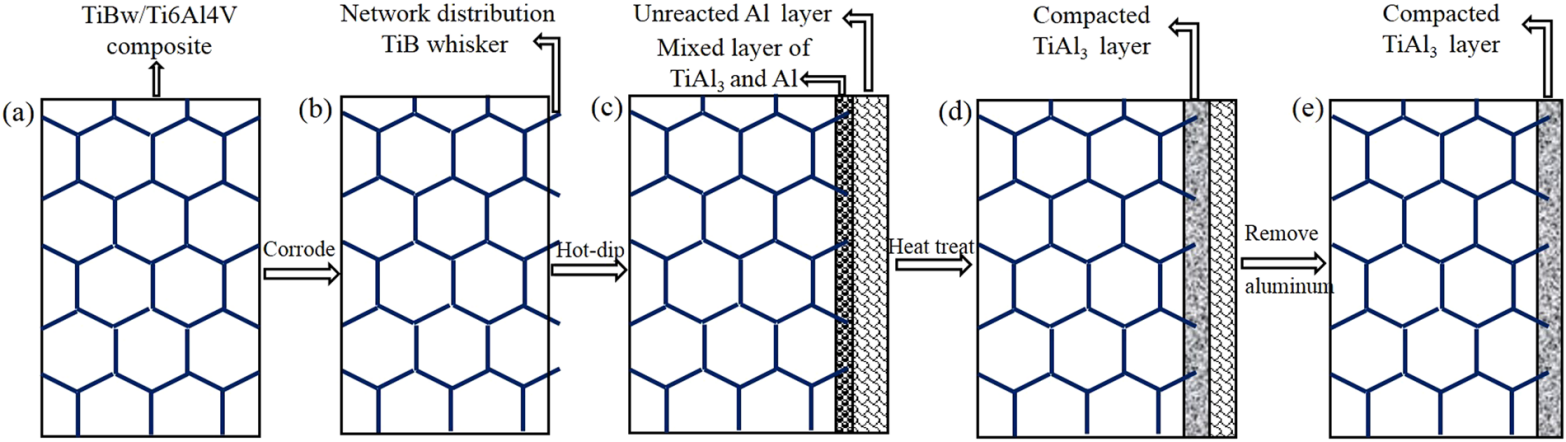
The schematic diagram of the TiAl_3_ coating fabrication process.

The substrate used for this study was the network structured TiBw/Ti6Al4V composites of TiB in 5% volume fraction fabricated by the reaction hot pressing sintering. The specimens which had dimensions of 10 mm × 10 mm × 3 mm were ground to 1000 grit with different grid sizes of SiC papers, and cleaned ultrasonically in ethanol, then dried immediately. As shown in Fig. 11(a) and (b), in order to increase the bonding strength of the coating, TiBw/Ti6Al4V composites were firstly corroded to expose the TiB whiskers in the acid solution of 5%HF and 15%HNO_3_ for 7–10 s, followed by rinsed in a stream of water and cleaned with ethanol ultrasonically, and then were dried.

In order to keep the surface of TiBw/Ti6Al4V composites from oxidizing in air, the specimens were immersed in 6%NaCl + 4KF% solutions at 353 K for 10 min, which lead a protection film on surface of the composite specimens. Furthermore, 50% KCl, 37.5% NaCl, 12.5% KF with mass ratio were covered in the aluminum melt in advance. The pretreated specimens were immersed in the molten aluminum at 1123 K for 10 min, and withdrawn from the aluminum melt and cooled in air (Fig. 11(c)). Because the coating with single step high temperature immersing is not compacted and controlled, therefore, the diffusion annealing treatment of the hot-dipped specimens were subsequently carried out in the muffle furnace at 923 K for 6 h and then cooled in the furnace to room temperature (Fig. 11(d)). The adherent aluminum material on the surface of the heat treated specimen was removed by immersing in the 15% NaOH solution (Fig. 11(e)).

The oxidation behaviors of the uncoated and coated specimens were examined by the cycle oxidation tests at 873 K, 973 K and 1073 K for 100 h, respectively. During the oxidation tests, all the specimens were heated in a muffle furnace at desired temperature in ambient air, then removed from the furnace every 10 h and air-cooled to room temperature. The specimens were placed in alumina crucible separately, thus the spalling oxide scales were also weighted. Their weight changes were measured at regular intervals using an electronic balance with a sensitivity of 0.1 mg.

Before and after the oxidation tests, the surface and cross-section morphologies of the specimens were characterized by the scanning electron microscopy in a SUPRA55 instrument equipped with energy dispersive spectroscopy (EDS). The phase compositions of the coatings were analyzed by the Empyrean X-ray diffraction (XRD) with Cu Kα radiation in a step of 0.02° and a range of 2θ from 20° to 90°.

### Data availability statement

The authors promise all of data in this paper are available.

## Conclusions

1. The compacted TiAl_3_ coating on the TiBw/Ti6Al4V composites with network structure was successfully fabricated by hot-dip aluminum and interdiffusion treatment. The inherited network structure of the TiAl_3_ coating can effectively reduce the thermal stress generated by the mismatch of thermal expansion coefficient between the coating and composite substrate, which avoids cracks appeared in the coating. Moreover, the spinning effect of the TiB whiskers can remarkably improve the bonding strength between the coating and the composite substrate.
2. Cycle oxidation tests of the coated and uncoated specimens showed the TiAl_3_ coating significantly improve the oxidation resistance of the composites, this is mainly due to the dense and continuous Al_2_O_3_ oxide scales and Al_2_O_3_ + TiO_2_ transition layer produced during the high temperature oxidation process.
3. The network structure was also inherited to the oxide scale during the oxidation process, which can effectively reduce thermal stress of the oxide scales and decrease the tendency of cracking even spalling about oxide scales, therefore, the oxide scale with network structure can provide good protection against the oxidation.
4. The interfacial reaction zone with layered structure of TiAl and TiAl_2_ was observed between the coating and the composite substrate, which can further decrease the thermal stress of the coating and avoid the cracking even spallation in the coating during the long-time oxidation process.
